# Self-Selecting Resistive Switching Scheme Using TiO_2_ Nanorod Arrays

**DOI:** 10.1038/s41598-017-01354-7

**Published:** 2017-05-18

**Authors:** Chi-Hsin Huang, Ta-Shun Chou, Jian-Shiou Huang, Shih-Ming Lin, Yu-Lun Chueh

**Affiliations:** Department of Materials Science & Engineering, National Tsing-Hua University, Hsinchu, 30013 Taiwan, ROC

## Abstract

In this study, the resistive switching scheme using TiO_2_ nanorod arrays synthesized by a large-scale and low-cost hydrothermal process was reported. Especially, the nonlinear *I–V* characteristics of TiO_2_ nanorod arrays with a nonlinearity of up to ~10, which suppress the leakage current less than 10^−4^ Acm^−2^, were demonstrated, exhibiting a self-selecting resistive switching behavior. It provides a simple pathway for integration of RRAM crossbar arrays without additional stacking of active devices. The mechanisms of the nonlinear resistive switching behaviors were discussed in detail. In addition, the maximum array numbers of 79 for self-selecting RRAM cells were estimated. The results demonstrate an opportunity of using the concept of self-selecting resistive switching characteristics in a single material, which offers a new strategy to tackle the sneak path issue of RRAM in the crossbar arrays structure.

## Introduction

Among the emerging nonvolatile memory (NVM) technologies, resistive switching random access memory (RRAM) utilizing resistive switching (RS) phenomena is one of the most promising candidates for next-generation nonvolatile memory due to the simplest device structure, the fastest switching speed, the highest stacking density, the lowest power consumption, the largest scalability, the lowest fabrication process cost and the strongest potential for fabricating multistate memories^1–4^. The resistive switching phenomena in the transition metal oxide, such as NiO^5^, TiO_2_^6, 7^, ZnO^8^, and Cu_2_O^9^ through two-dimension (2D) metal-insulator-metal (MIM) thin film structures with excellent memory performances has been intensively investigated and demonstrated, previously.

An effective method to increase memory integration density can be realized *via* three-dimensional crossbar architecture arrays (Fig. S1a), with which the smallest possible cell size of 4F^2^ (F = minimum feature size) for high-density nonvolatile memory applications can be achieved^10^. However, an inherent problem, namely a sneak path issue, for which the leakage current flows through neighboring RRAM devices of the crossbar arrays, occurs^11^. The designated cell with high resistance state (HRS), while the undesignated neighboring cells are in the low resistance state (LRS), may generate parasitic paths in the crossbar array with applied external voltage between the word-line (top) and the bit-line (bottom) (Fig. S1b). Consequently, the total read current is much higher than the accessed current due to the sneak current through the neighboring cells, leading to not only unnecessary power consumption but also a misreading problem. Note that the readout margin is significantly decreased as the crossbar array size increases, which eliminates the scalability of memory in the crossbar arrays^11, 12^. Therefore, it is necessary to find the method to overcome the sneak current issue and achieve the excellent scalability. In order to access any device randomly without reading interference between neighboring cells, each memory element must be connected with a selecting element to prevent sneak path current problem (Fig. S1c). Using a transistor, which occupies a 6–8F^2^ size as the selecting device to overcome the sneak current in a crossbar array, normally limits stacking capability of achieving 4F^2^ integration densities^13^. Alternatively, passive crossbar arrays utilizing one diode-one resistor (1D1R)^12, 14, 15^, one selector-one resistor (1S1R)^16, 17^ or complementary resistive switches (CRS)^11, 18–20^ with the *I–V* nonlinearity characteristics provide effective ways to overcome the sneak path issue. Either the combination of the selecting device or the concept of CRS makes the fabrication of the memristor more complicated with high manufacture cost and low stacking density.

To reduce the dimension of devices and achieve a high packing density with the improved device performance, finding out suitable device configuration through nanostructures associated with unique electrical properties is a useful way to explore the underlying resistive switching mechanisms in nanoscale. The resistive switching characteristics of nanostructured materials, such as ZnO NW^21–23^, NiO NW^24–26^, CuO_x_ NW^27, 28^, Co_3_O_4_ NW^29^, Zn_2_SnO_4_ NW^30^ and gold-Ga_2_O_3_ NW^31^, have attracted great attention because of a simple fabrication process with the low power consumption and high density storage. In addition, integration of two oxide layers into bilayer oxide configuration as an insulating layer exhibits a selective functionality with an engineered nonlinearity^32–34^. Moreover, the promising material, such as VO_x_, with self-selecting resistive switching performance for crossbar memory arrays was demonstrated^35^. Such unique property offers a simple way for practical application without additional device steak.

Although various TiO_2_ thin film devices have been widely evaluated as a resistive switching material for RRAM^6, 7, 36^, the resistive switching characteristics of TiO_2_ nanorod arrays (NRs) structure have seldom been discussed. In this regard, the self-selecting resistive switching characteristics of TiO_2_ nanorod grown on a fluorine-doped tin oxide (FTO) by a hydrothermal method are investigated for the first time^37^. Using Pt/TiO_2_ NRs/Pt to fabricate RRAM device, the *I–V* characteristics with nonlinearity up to ~10 were successfully demonstrated. The characteristics of Pt/TiO_2_ NRs/Pt were measured to suppress the leakage current <3.7 V, showing that the leakage current in the TiO_2_ NRs device was significantly limited within 10^−4^ Acm^−2^. The mechanisms of the switching behavior and nonlinear electrical properties were investigated and discussed in detail. The concept of nonlinear *I–V* characteristics in single material offers the new strategy to overcome the sneak path problem in the crossbar arrays structure.

## Results and Discussion

**Figure 1:**
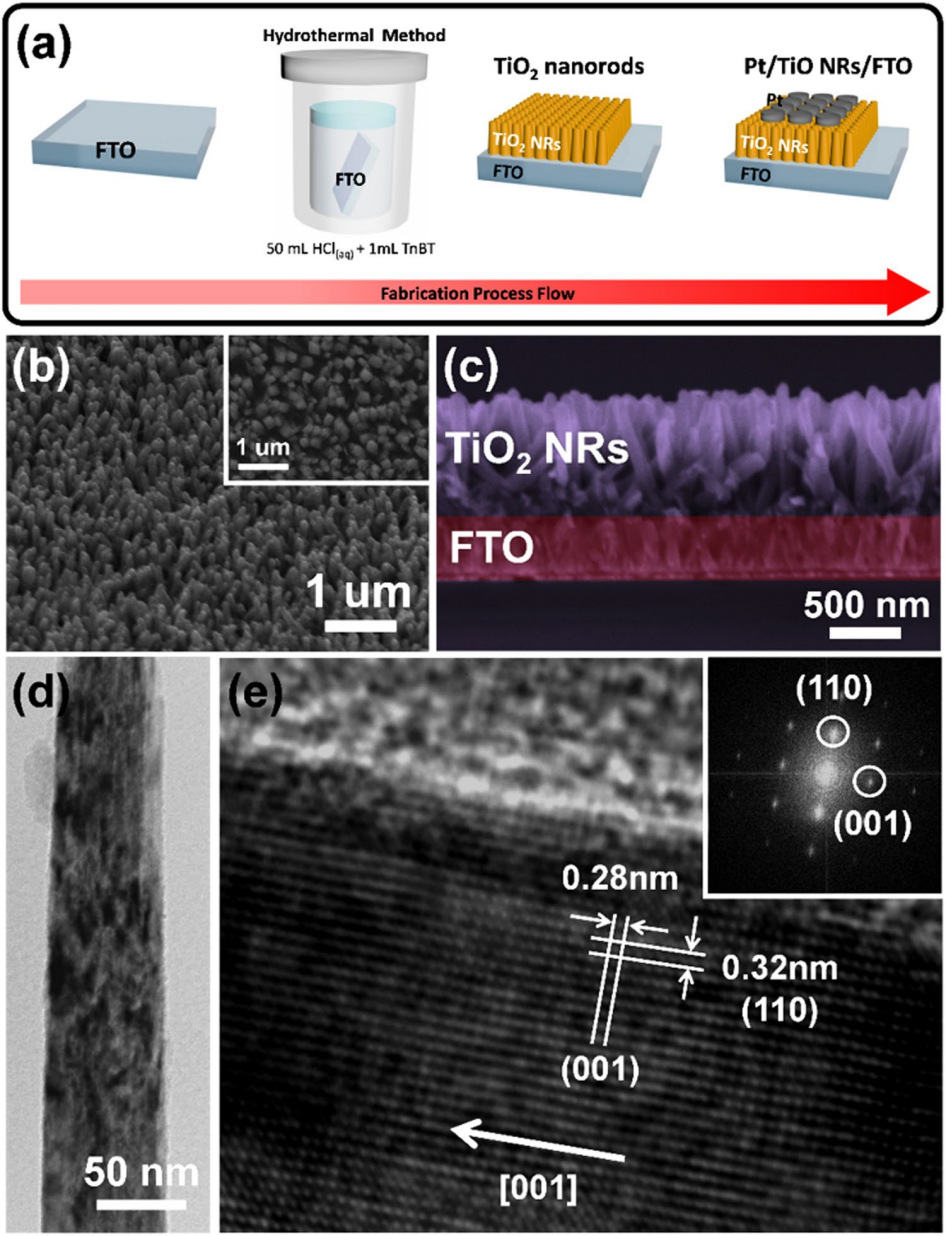
(**a**) Schematics of the fabrication processes of TiO_2_ NR arrays and Pt/TiO_2_ NRs/FTO device. (**b**) Tilted SEM image of TiO_2_ nanorod arrays. The inset shows a top-view SEM image of TiO_2_ nanorod arrays. (**c**) Cross-sectional SEM image of vertically aligned TiO_2_ nanorod arrays with a length of ~700 nm grown on FTO substrate. (**d**) A low-magnification TEM image of TiO_2_ nanorod. (**e**) HRTEM image of the TiO_2_ nanorod. The inset shows the diffraction pattern extracted by fast Fourier transform (FFT), confirming a single crystalline structure of the TiO_2_ nanorod.

Fig. 1(a) shows schematics of rutile TiO_2_ nanorod arrays grown on a FTO glass substrate by a previously reported hydrothermal method^37^. TiO_2_ NR arrays were hydrothermally grown on the fluorine-doped tin oxide (FTO) substrate in 50 mL of aqueous hydrochloric acid (the ratio of DI water to 37% HCl is 1:1) and 1 mL of titanium (IV) test-n-butoxide (TnBT) in a Teflon-lined stainless steel autoclave (125 ml volume) at 150 °C for 3 hours, followed by heat treatment in air at 350 °C for 3 hours to increase the crystallinity of TiO_2_ nanorods and improve their contact to the substrate. Fig. 1(b) and inset show a corresponding field-emission scanning electron microscopy (FESEM) image of one-dimensional, high-density and well-aligned TiO_2_ nanorod arrays on the FTO substrate. The corresponding cross-sectional SEM image as shown in Fig. 1(c) clearly confirms the diameters of TiO_2_ nanorods are in the ranges of 30~200 nm and the typical nanorod lengths of ~700 nm. The tetragonal rutile structure of TiO_2_ nanorods was confirmed by X-ray diffractometer (XRD)with ICDD-PDF No.01-088-1175 where two major peaks at planes of (101) and (002) can be indexed as shown in Fig. S2. Furthermore, Fig. 1(d) shows a low magnified transmission electron microscopy (TEM) image of a TiO_2_ nanorod. The corresponding high-resolution TEM (HRTEM) image as shown in Fig. 1(e) reveals a single crystalline nature. Inset in Fig. 1(e) shows a selected area diffraction (SAED) pattern, also confirming the single crystalline nature of the TiO_2_ nanorod with the rutile phase. Two internal spacings of 0.32 and 0.28 nm, which are consistent with the d-spacings of (110) and (001) planes of the rutile TiO_2_, were indexed. As a result, the growth direction along [001] can be confirmed. Moreover, peaks at 265, 429 and 606 cm^−1^, corresponding to the multi-photon process, E_g_ and A_1g_^38^ were observed in the Raman spectra as shown in Figure S3, providing another solid evidence of rutile TiO_2_ nanorods grown on the FTO substrate.

**Figure 2:**
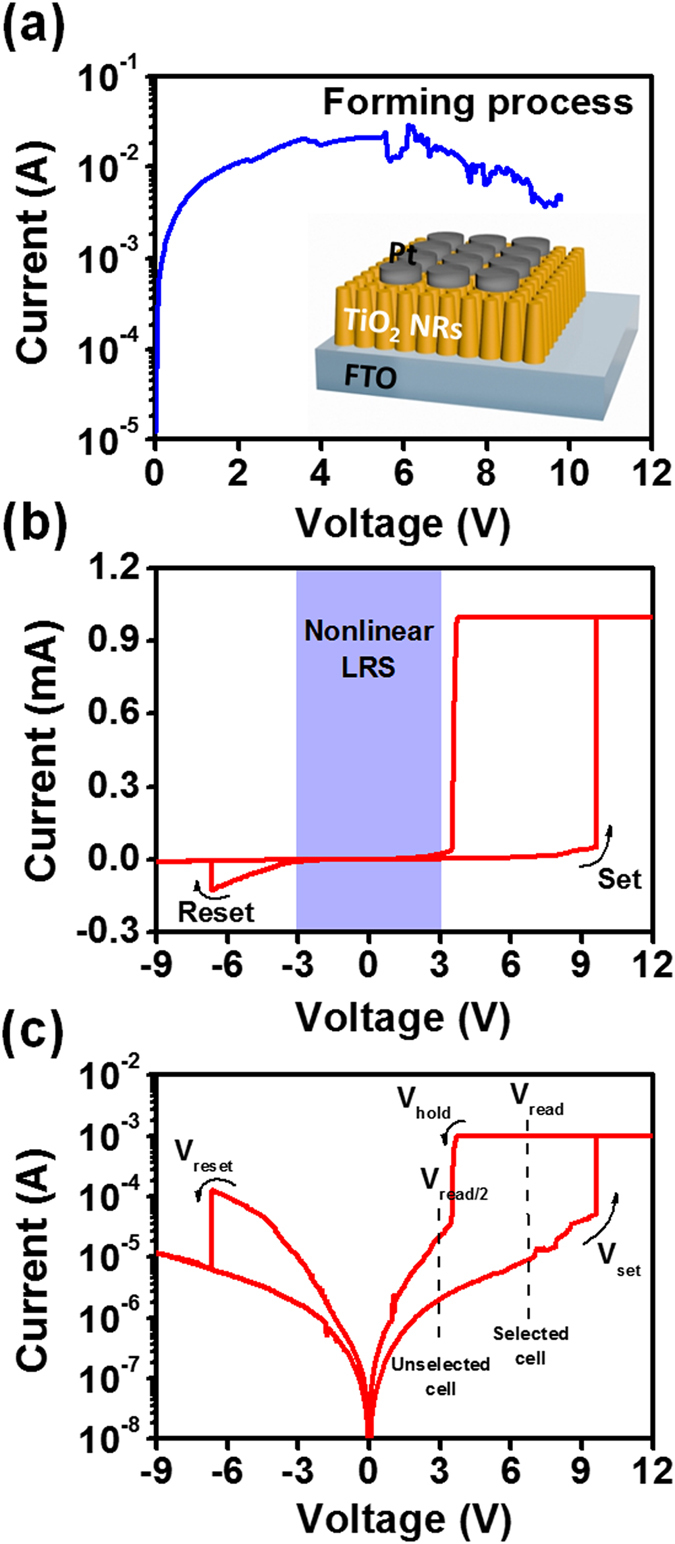
(**a**) Typical *I-V* characteristics of a forming process for Pt/TiO_2_ NRs/FTO device. The inset shows a schematic of a Pt/TiO_2_ NRs/FTO device. (**b**) Typical resistive switching behavior of Pt/TiO_2_ NRs/FTO device in linear scale. (**c**) Typical resistive switching behavior of Pt/TiO_2_ NRs/FTO device in semi-log scale.

For resistive switching characteristics measurements, a Pt thin film with a thickness of 100 nm as the top electrode was deposited on TiO_2_ nanorod arrays with ~700 nm in length by the rf-magnetron sputtering at room temperature while the FTO substrate was used as the bottom electrode (BE) as shown in inset of Fig. 2(a). Note that the diameter of the Pt electrode is fixed at 200 nm by a metal shadow mask. *I–V* characteristics of the Pt/TiO_2_ NRs/FTO memory device were studied by DC voltage sweep measurements where the bias voltages were applied to the top electrode (TE) with the bottom electrode (BE) grounded, and the pristine device with a resistance of ~167 Ω from *I–V* curve was measured. Before resistive switching phenomena are achieved, an irreversible “Forming process” has to be applied. A gradual decrease of the resistance was observed as shown in Fig. 2(a) after applying a continuous DC voltage sweep to ~9.8 V on the top electrode. The resistive switching of from low resistance state (LRS) to high resistance state (HRS) instead of from HRS to LRS in the forming process was found, which is different from the general forming process in RRAM device^39^. (Forming process of another device, Fig. S4) Fig. 2(b,c) represent typical I–V characteristics of a Pt/TiO_2_ NRs/FTO device with linear and semi-log plots, respectively. By applying a positive bias from 0 V to 12 V with a compliance current (CC) of 1 mA, an abrupt increase of current could be observed at 9.6 V, which is called a SET voltage (V_set_). The resistance of the device was changed from the HRS to LRS during this operation, namely the “SET process”, as shown in Fig. 2(b,c). On the other hand, with a decrease in applied voltage from 12 to 0 V, the suddenly decreased current at 3.7 V can be found, which is called a hold voltage (V_hold_) as the electrical property of TiO_2_ returns back to the insulating state. It could be observed that there was a significantly increased resistance, leading to current suppressed at a low voltages region. When we sweep to the negative bias, the resistance of the device switching to HRS at ∼−6.65 V (RESET voltage, V_reset_) was observed, which is called the “RESET process”. At voltages below the V_hold_, the limited current can be reached, namely nonlinearity resistive switching, which can be accessed by applying an appropriate reading voltage (V_read_) from V_hold_to V_set_. Write operation can be performed at voltages >3.7 V while erase operations can be performed at voltages <−6.7 V. (*I-V* curve of another device are shown in Figure S5.) The selecting operation of the nonlinearity resistive switching found in Pt/TiO_2_ NRs/FTO device can be used to all of the unselected cells, which can be considered as the worst case with the *N* × *N* cross-point arrays in the LRS as shown in Fig. S1 ^40^. At this half-biased scheme, V_read_ was effectively applied to the selected cell, whereas V_read_/2 and −V_read_/2 were imposed to unselected cells (*N* + *N* − 2) connected the applied word line and bit line, mainly contributing to the sneak path current^40^. In our device, V_read_ and V_read/2_ at 6 and 3 V were assumed. The LRS current or the leakage current of the unselected cells (unselected cells are biased at V_read/2_) was suppressed while keeping V_read_/2 below 3.7 V (V_hold_), showing that the leakage current was significantly limited within 10^−4^ Acm^−2^. The nonlinearity is about 10 as an important factor for improving the readout margin in cross-point arrays, which is defined as I(V_read_)/I(V_read/2_), where V_read_ is the full-read voltage and V_read/2_ is the half-read voltage. The nonlinearity resistive switching in the TiO_2_ NRs device offers the selecting function, which could decrease reading interference between neighboring cells, thus improving the crossbar arrays size. In addition, the length difference will also affect resistivity of the nanorod. If the resistivity of the NRs layer is too high, the forming process needs a larger bias to the following operation, even no resistive switching behavior. If the NRs layer resistivity is too low, the NRs layer may act as a simple metallic contact. Therefore, TiO_2_ NRs with the length of ~700 nm in the current study can be a suitable length with the good performance memory operation.

**Figure 3:**
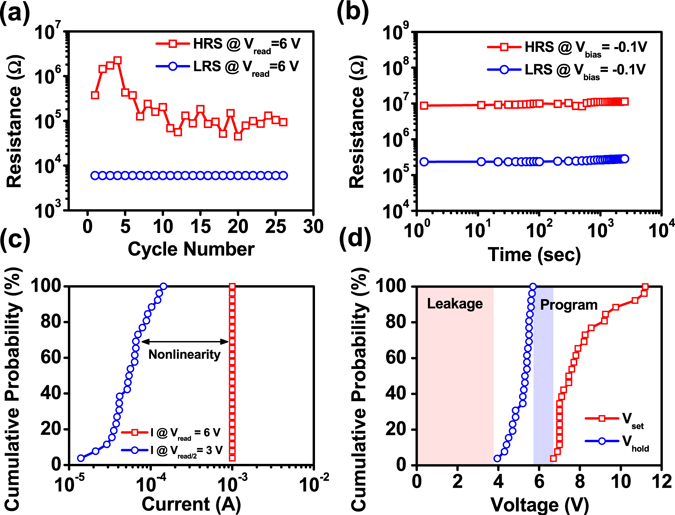
(**a**) Endurance test of the Pt/TiO_2_ NRs/FTO device under DC switching sweep mode at a read voltage of 6 V. (**b**) Retention tests of the Pt/TiO_2_ NRs/FTO device under a continuous read voltage stress of −0.1 V. (**c**) Cumulative distribution of current in LRS at V_READ_ and V_READ/2_ for the Pt/TiO_2_ NRs/FTO device. (**d**) Cumulative distribution of V_SET_ and V_hold_ for the Pt/TiO_2_ NRs/FTO device.

Endurance and retention tests were conducted in order to investigate the reliability of the Pt/TiO_2_ NRs/FTO device. Obtained by repetitive ON/OFF sweeping operation, the endurance cycling test results are shown in Fig. 3(a). The typical resistances of the ON-state (LRS) and the OFF-state (HRS) on the order magnitudes of 10^4^ and 10^5^~10^6^ are obtained using the read voltage at 6 V. With a R_OFF_/R_ON_ ratio of approximately 10~10^2^, the memory device has a sufficient window for efficient memory applications. The resistive switching in TiO_2_ NRs devices is reproducible with cyclic tests of ~25 cycles. Until now, the cycle performance is still limited by lack of enough vacancy migration to switch device from HRS to LRS under the high bias operation, which can be expected to be improved by decreasing the length of nanorods with optimized crystallinity. The retention property of memory was conducted to evaluate the data storage ability at room temperature, and the obtained results using the read voltage at −0.1 V are shown in Fig. 3(b). Distinctly, the memory shows good retention characteristics up to 10^3^ sec without obvious degradation in both the HRS and LRS. The cumulative probability distributions of current in LRS at V_read_ and V_read_/2 in the TiO_2_ NRs device were measured as shown in Fig. 3(c). The device maintains the nonlinear performance during the switching cycle, namely self-selecting resistive switching property. Figure 3(d) shows cumulative probability distributions of V_set_ and V_hold_ obtained from *I-V* curves of TiO_2_ NRs device. For V_hold_ < 3.7 V, the total current of the device is ensured being limited, suppressing the leakage current of the device. Note that the device can be programmed, and the stored information can be read by applying an appropriate reading voltage between V_set_ and V_hold_. The experimental results from Fig. 3(c,d) indicate that the switching performances are reproducible from cycle to cycle.

**Figure 4:**
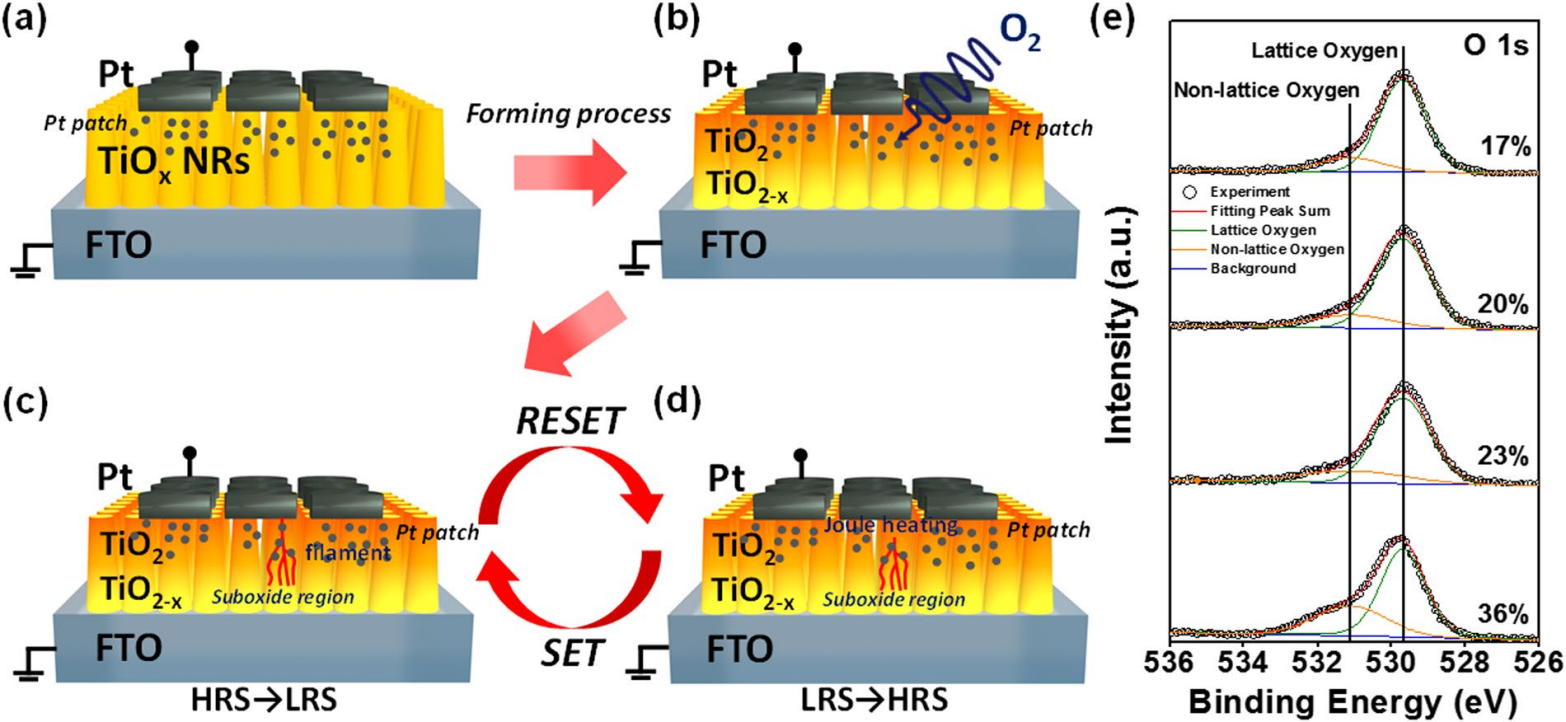
Schematics of nonlinear resistive switching mechanism: (**a**) In the initial state, (**b**) after forming process, (**c**) after the set process and (**d**) after resetting process. (**e**) XPS spectra at O 1 s core-level of TiO_2_ NRs from top to bottom regions of TiO_2_ NRs.

The possible model to explain the observed nonlinear resistive switching phenomenon were proposed as shown in Fig. 4. In the “Forming process”, an amount of heat generated by the Joule heating flowing into TiO_2_ NRs can easily trigger an oxidation reaction, making oxygen-deficient TiO_2_ NRs more oxidized after applying a large bias with a high current density (Fig. 4a). The energy dispersive spectrum (EDS) results of pristine TiO_2_ NR show that atomic compositions of Ti and O are of 37.2 and 62.8 at %, respectively, confirming the oxygen-deficient in TiO_2_ NR with the O/Ti ratio of ~1.67 as shown in Fig. S6. The geometry of nanorod arrays provides a large area surface to adsorb O_2_ molecules to accelerate the oxidation process, leading to an increase in resistance of TiO_2_ NRs. Otherwise, the O_2_ molecules adsorbed on the defective area of TiO_2_ NRs surface serve as the traps for charged carriers to form chemisorbed oxygen adatoms and increase the surface potential, leading to the decrease in the conductivity of TiO_2_ NRs surface^41^. The diameter of TiO_2_ NRs becomes larger, resulting in the bottom of nanorods being wider than the top region when the nanorods grow taller from bottom to top and ultimately touch each other to form a continuous film at the bottom (see the Fig. 1c)^37^. In this way, the porosity of TiO_2_ NRs film near top electrode is higher than that near bottom electrode. The different porosities (the different sizes of the surface area) to adsorb O_2_ and the migration of oxygen ions under the positive bias result in the formation of the TiO_2-x_ suboxide region (TiO_2_ layer with oxygen-deficient region) layer near the bottom FTO electrode (cathode, grounded electrode) and TiO_2_ fully-oxide region (TiO_2_ layer with oxygen-rich region) near the top Pt electrode (anode, the electrode on which the voltage was applied) (Fig. 4b). During the SET process as shown in Fig. 4(c), oxygen vacancies diffuse from the anode (the electrode on which the voltage was applied) to cathode (grounded electrode) along with nanorod surface and form metallic filaments to connect the top electrode and the suboxide region, and switch device from HRS to LRS. Additionally, the oxygen-deficient TiO_2-x_ suboxide region serves as a supplementary reservoir of oxygen vacancies to connect conducting filaments. Simultaneously, electrons can be injected from cathode to anode. The Joule heating effect by electron injection should provide a thermal energy to drive ionic migration^42, 43^. Note that the filaments usually have larger size near the bottom electrode and small size near the grounded electrode^42, 44^. The oxygen-deficient TiO_2-x_ suboxide region plays an important role, resulting in non-linearity *I-V* phenomena. Numbers of non-stoichiometric Ti suboxides (Ti_n_O_2n-1_, so-called Magne´li phase), including Ti_2_O_3_, Ti_3_O_5_, Ti_4_O_7_, Ti_5_O_9_^45^, have been known to demonstrate the gradual transition from metal to insulator^33^. In our case, non-linearity *I-V* characteristics in the device may be attributed to a gradual transition of suboxide phase in TiO_2-x_ suboxide region after the formation of filaments during the SET process. In addition, the deposition of Pt electrode is also deposited on the surface of NRs due to the porosity of TiO_2_ NRs film, which would form a metal patch on the nanorod surface and this patch distribution is the difference from top and bottom of NRs. Top of nanorods has more metal patch to increase the conductivity by assisting connection of the filaments in LRS while the bottom of nanorods seldom has metal patch owing to lower porosity of TiO2 NRs film. Metal patch distribution and oxidation difference from top and bottom of NRs would result in the Schottky barrier height, suppressing the current in LRS. Therefore, the current of LRS would be suppressed in the low bias. In the RESET process, a significant amount of heat generated by the Joule heating interrupts the local filaments when the high current density flows into the metallic filaments (Fig. 4d)^42, 43^. Simultaneously, Joule heating also provides energy to the thermochemical reaction, causing the migration of oxygen vacancies (oxygen ions). The positions for the rupture of the filaments would normally form near the top electrode owing to much thinner filament sizes. To corroborate the existence of the non-stoichiometric Ti suboxides region nearby the bottom FTO electrode, X-Ray photoelectron spectroscopy (XPS) at the O 1 s core-level was used to identify the oxygen distribution of TiO_2_ NRs from top to bottom regions after the resistive switching operation as shown in Fig. 4(e). From the peak fitting deconvolution analysis, a distinct peak at 531.1 eV^46^ owing to non-lattice oxygen in oxygen deficient TiO_x_ indicates the presence of unbonded oxygen molecules (oxygen interstitials) and oxygen vacancies, while a peak at 529.7 eV^46^ related to the lattice oxygen in stoichiometric TiO_2_ was found. The higher percentage of non-lattice oxygen near the TiO_2_ NRs bottom side distinctly confirms the existence non-stoichiometric Ti suboxides region nearby the bottom FTO electrode.

**Figure 5:**
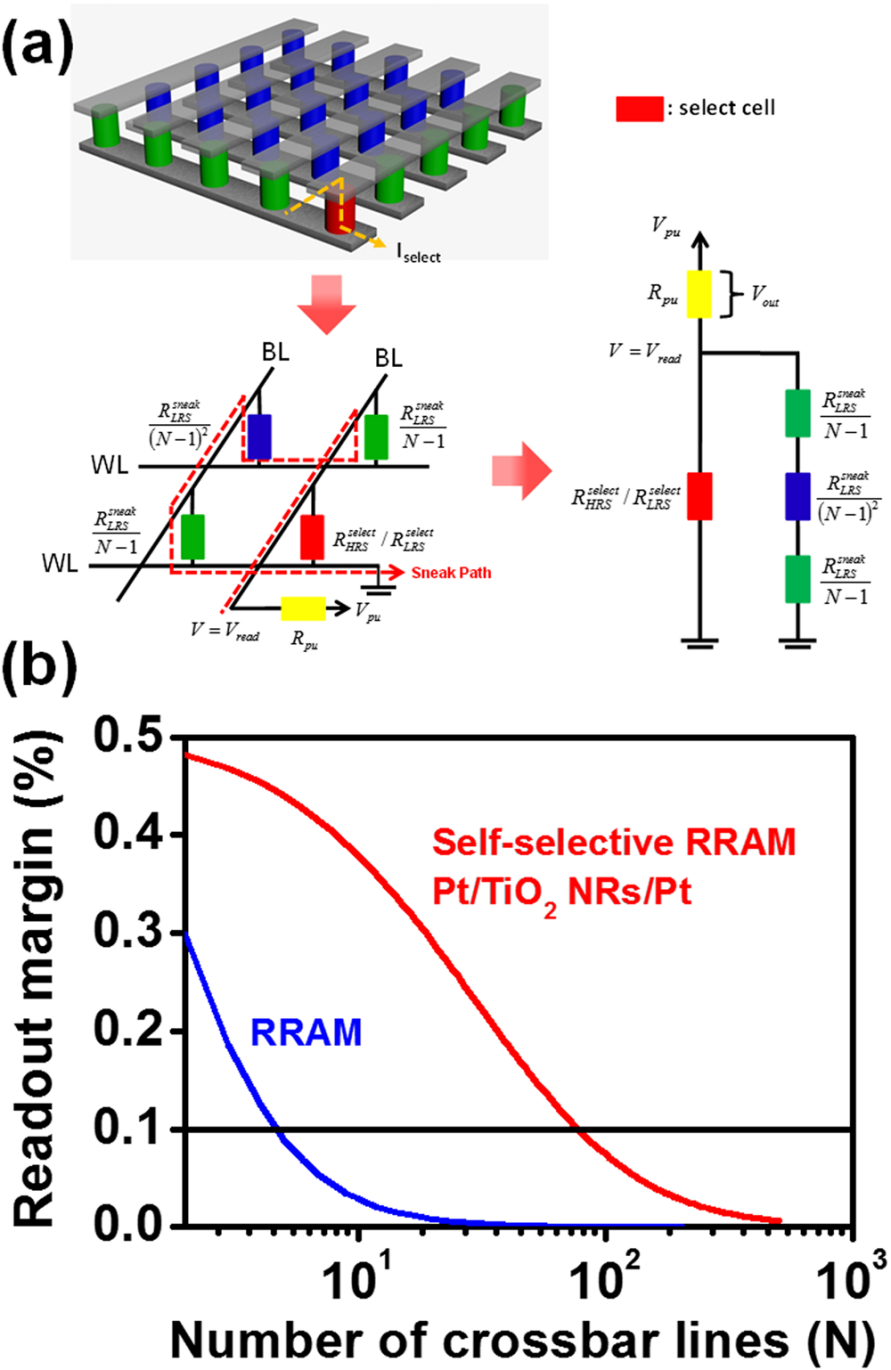
(**a**) A schematic of *N* × *N* crossbar arrays and the corresponding equivalent circuit of crossbar arrays using one bit-line and pull-up read scheme, with which the red dotted line denotes the sneak path. The misreading current path (sneak path) in *N* × *N* crossbar arrays occurs where all bits are at LRS except the red cell is selected. The green regions are parallel resistor networks, namely bits on selected BLs and bits on selected WLs. The blue regions are bits on unselected WLs and BLs. (**b**) The dependence of normalized read voltage margin ΔV/V_pu_ with the crossbar line number (N) at read voltages of 0.1 and 6 V for the general RRAM device and the self-selecting resistive switching Pt/TiO_2_ NRs/FTO device.

The major advantage of the self-selecting resistive switching behavior of TiO_2_ NRs compared with general RRAM elements is the suppression of sneak paths to achieve large passive crossbar arrays. To estimate the maximum crossbar array density of the resistive switching behaviors of TiO_2_ NRs, two passive crossbar arrays (*N* rows × *N* columns) using general RRAM cells (1R)^12^ and self-selecting RRAM cells based on TiO_2_ NRs with the worst read scheme were evaluated where the crossbar array can be simplified as the equivalent circuit in Fig. 5(a) ^40^. The read margin (ΔV_out_) normalized to pull-up voltage (V_pu_) as an important parameter of estimating the cross array density can be calculated by the Kirchhoff equation given by refs 40 and 47 (Detail calculation, please read the supporting information)

$$\frac{{{\rm{\Delta }}{\rm{V}}}_{\mathrm{out}}}{{{\rm{V}}}_{{\rm{pu}}}}=\frac{{{\rm{V}}}_{{\rm{out}},{\rm{HRS}}}}{{{\rm{V}}}_{{\rm{pu}}}}-\frac{{{\rm{V}}}_{{\rm{out}},{\rm{LRS}}}}{{{\rm{V}}}_{{\rm{pu}}}}=\frac{{{\rm{R}}}_{{\rm{pu}}}}{[{{\rm{R}}}_{{\rm{HRS}}}^{{\rm{select}}}\parallel {{\rm{R}}}_{{\rm{sneak}}}]+{{\rm{R}}}_{{\rm{pu}}}}-\frac{{{\rm{R}}}_{{\rm{pu}}}}{[{{\rm{R}}}_{{\rm{LRS}}}^{{\rm{select}}}\parallel {{\rm{R}}}_{{\rm{sneak}}}]+{{\rm{R}}}_{{\rm{pu}}}}$$

Where V_out_, R_select_, R_sneak_ and R_pu_ represent the voltage across the pull-up resistor, parameters of selected cell resistance, sneak path resistance and connective resistance in the measured system, respectively. Note that the sneak resistances can be given by ${{\rm{R}}}_{{\rm{sneak}}}=\frac{2{{\rm{R}}}_{{\rm{LRS}}}^{{\rm{sneak}}}}{{\rm{N}}-1}+\frac{{{\rm{R}}}_{{\rm{LRS}}}^{{\rm{sneak}}}}{{({\rm{N}}-1)}^{2}}$. As a result, the dependence of ΔV_out_/V_pu_ on the crossbar number (*N*) in 1R and self-selecting RRAM can be shown in Fig. 5(b). Notably, the read margin decreases as the crossbar numbers increase for both the general RRAM cells and the self-selecting RRAM cells. Therefore, the maximum *N* numbers of 4 and 79 for the general RRAM cells and the self-selecting RRAM cells could be achieved by taking ΔV/V_pu_ of 0.1 (10%) into account, respectively. Clearly, the improved readout margin in the cross-point array of the self-selecting RRAM cells compared to the general RRAM device due to the nonlinearity of Pt/TiO_2_ NRs/FTO configuration can be confirmed. In the future, more works will be clearly needed to improve the self-selecting memory performance, and to increase the ratio of R_sneak_/R_LRS_^24^ for enlarging the crossbar array numbers (Fig. S7).

## Conclusions

To summarize, the resistive switching characteristics of TiO_2_ nanorod system synthesized by a large-scale and low-cost hydrothermal method has investigated. As a proof of concept, the nonlinear *I–V* characteristics of TiO_2_ nanorod arrays was measured to suppress the leakage current less than 10^−4^ Acm^−2^, demonstrating the self-selecting resistive switching with nonlinearity up to ~10 for crossbar memory arrays application. The unique property offers a simple way for practical crossbar arrays application without additional device steak. The mechanism of the nonlinear resistive switching behavior was discussed in detail. The concept of self-selecting resistive switching in single material offers the new strategy to overcome the sneak path issue for RRAM in the crossbar arrays structure.

## Methods

### Synthesis of Rutile TiO_2_ NR arrays

Rutile TiO_2_ NR arrays were hydrothermally grown on the fluorine-doped tin oxide (FTO) substrate in 50 mL of aqueous hydrochloric acid (the ratio of DI water to 37% HCl is 1:1) and 1 mL of titanium(IV) test-n-butoxide (TnBT) in a Teflon-lined stainless steel autoclave (125 ml volume) at 150 °C for 3 hours. After the reaction, the sealed autoclave was cooled down to room temperature slowly. After the NR are grown, the substrate was rinsed with deionized water and baked at 80 °C. Finally, the sample was annealed in air at 350 °C for 3 hours to increase the crystallinity of TiO_2_ nanorods and improve their contact to the substrate.

### Device Fabrications

After the synthesis of TiO_2_ NR arrays, the Pt as the top electrode with a thickness of 100 nm was deposited on the TiO_2_ NR arrays *via* rf-magnetron sputtering at room temperature. The Pt electrodes of diameters 200 were fabricated *via* a metal shadow mask.

### Characterizations

A field emission scanning electron microscope (FE-SEM, JSM-6500F, JEOL), operating at 15 kV and a field emission transmission electron microscope (FE-TEM, JEM-3000F, JEOL) equipped with an energy dispersion spectrometer (EDS), operating at 300 kV were used to study surface morphologies and microstructures. X-ray diffraction spectrometer (Shimadzu XRD 6000, Cu Kα radiation with a wavelength of 0.154 nm) and Raman microscope (Horiba Jobin Yvon LabRam HR800 with an excitation wavelength of 632.8 nm) was performed to characterize crystal structure. The resistive switching characteristics of the fabricated devices were investigated using a Keithley 4200 semiconductor parameter analyzer in voltage sweeping mode at room temperature. All of the operation voltages were applied on the top Pt electrode, and the FTO as the bottom electrode was grounded. The chemical bonding states were characterized by X-ray photoelectron spectroscopy (XPS, ULVAC-PHI, PHI Quantera SXM).

## Electronic supplementary material

Supplementary Information
