# Supplementary material for: Self-Selecting Resistive Switching Scheme Using TiO2 Nanorod Arrays
Source: Sci Rep. 2017 May 18;7:2066. doi: 10.1038/s41598-017-01354-7 (PMC5437035; doi:10.1038/s41598-017-01354-7)
Supplement: Supplementary file 1 — Supplementary Information [file 41598_2017_1354_MOESM1_ESM.rtf]

Supplementary Information
Self-Selecting Resistive Switching Scheme Using TiO2 Nanorod Arrays
Chi-Hsin Huang, Ta-Shun Chou, Jian-Shiou Huang, Shih-Ming Lin and Yu-Lun Chueh†*
Department of Materials Science & Engineering, National Tsing-Hua University, Hsinchu 30013, Taiwan, ROC.
E-mail: ylchueh@mx.nthu.edu.tw


Figure S1 (a) Schematic diagram of crossbar memory array structure whose one bit cell of the array consists of a memory element and a switch element between conductive lines on top (bit line) and bottom (word line). (b) Reading interference in an array consisting of 2×2 cells without switch elements. (c) Rectified reading operation in an array consisting of 2×2 cells with switch elements.


Figure S2  XRD spectra of the TiO2 nanorod arrays.


Figure S3  Raman spectra of the TiO2 nanorod arrays.


Figure S4 The I-V curve of forming process for another device.


Figure S5 (a)(b) I-V curves of the TiO2 NRs devices measured on other two devices 


Figure S6  The energy dispersive spectrum (EDS) of the TiO2 nanorod arrays.


Figure S7 The dependence of normalized read voltage margin ÄV/Vpu with the crossbar line number (N) at read voltages for different Rsneak/RLRS/RHRS, showing that the ratio between Rsneak/RLRS plays the major role for enlarging the crossbar array numbers. The crossbar array numbers increase with the ratio of Rsneak/RLRS increasing.

Calculation of crossbar array sizes in read margin for 1R and 1SR devices
To estimate the maximum array density, the worst read scheme was used by assuming two crossbar arrays, namely the general RRAM (1R) elements, and the self-selective RRAM (1SR) elements, respectively. Regarding a N×N crossbar arrays with the worst read scheme for the reading of the crossbar array.1 This scheme allows only the bit-line (BL) and word-line (WL) connected to the selected cell, respectively. The worst case for reading the N×N crossbar arrays is expressed as follows: When reading an LRS-cell and the line resistance are negligible, the crossbar output voltage degrades as much as possible. This is a case if all unselected cells are LRS. When reading a word (one row), the measurable normalized read voltage margin ÄVout/Vpu is an important parameter.1 

A read scheme where a bit lines are pulled up can be considered.1, 2 The voltage across the pull-up resistor (Vout) between the HRS and the LRS in the selected bit could be distinguishable when a reading voltage (Vread) is applied to the selected bit-line. Although the current flows through the selected bit (Iselect), a sneak current also flows through unselected bits (Isneak). Consequently, the Vout is dependent of the Iselect and Isneak. Since the RLRS /(N-1) is substantially higher than RLRS /(N-1) along the sneak path, the voltage across the two similar components, RLRS /(N-1) is approximate to Vread/2.2 In this way, only Vread/2 would drop across the unselected bits on the selected word/bit lines. To achieve the highest ON/OFF ratio, the Vread was set to 0.1 V and 6 V for 1R and self-selecting RRAM, respectively. Table S1 summarizes extracted parameters for calculation of crossbar array numbers by solving the Kirchhoff equation to achieve the read margin (ÄVout) normalized to pull-up voltage (Vpu). The corresponding equivalent circuit of the crossbar is shown in Figure 5(a) where sneak resistances, effective sensible resistance values at HRS and LRS and resulting measurable normalized read voltage margin (ÄVout) could be given: 
The sneak resistances:

The effective sensible resistance values at HRS and LRS:


The resulting measurable normalized read voltage margin (ÄV):

Where the Rselect, Rsneak and Rpu represent parameters of selected cell resistance, sneak path resistance and connective resistance in measured system, respectively. The read margin decreased as increasing of the crossbar-line number (N) for both 1R and 1S1R devices. Therefore, the maximum values of N with at least 10 % read margin for general RRAM and self-selective RRAM devices were 4 and 77, respectively. This demonstrates that Pt/TiO2 NRs/FTO the device with self-selective characteristics could increase the crossbar array density. 

Table S1. Extracted parameters for the calculation maximum array size of general RRAM and self-selective RRAM devices
	Vread (V)				N with
>10 % ÄV/Vpu	
1R	0.1	5053	49	48	4	
1S1R	6	876757.16	6000	135661.89 	79	


REFERENCES
1. Flocke, A. & Noll, T. G. in Solid State Circuits Conference, 2007. ESSCIRC 2007. 33rd
  European.  328-331.
2. Jiun-Jia H, Yi-Ming T, Wun-Cheng L, Chung-Wei H, Tuo-Hung H. One selector-one                
  resistor (1S1R) crossbar array for high-density flexible memory applications. In: Electron   
  Devices Meeting (IEDM), 2011 IEEE International (ed^(eds) (2011).
